# Passively sensing smartphone use in teens with rates of use by sex and across operating systems

**DOI:** 10.1038/s41598-024-68467-8

**Published:** 2024-08-03

**Authors:** Jordan D. Alexander, Janosch Linkersdörfer, Katherine Toda-Thorne, Ryan M. Sullivan, Kevin M. Cummins, Rachel L. Tomko, Nicholas B. Allen, Kara S. Bagot, Fiona C. Baker, Bernard F. Fuemmeler, Elizabeth A. Hoffman, Orsolya Kiss, Michael J. Mason, Tam T. Nguyen-Louie, Susan F. Tapert, Calen J. Smith, Lindsay M. Squeglia, Natasha E. Wade

**Affiliations:** 1 University of Minnesota, Minneapolis, USA; 2 Department of Psychiatry, University of California, San Diego, 9500 Gilman Drive, MC 0405, La Jolla, CA 92093 USA; 3 University of Michigan, Ann Arbor, USA; 4 University of Wisconsin-Milwaukee, Milwaukee, USA; 5 California State University, Fullerton, USA; 6 Medical University of South Carolina, Charleston, USA; 7 University of Oregon, Eugene, USA; 8 University of California, Los Angeles, Los Angeles, USA; 9 SRI International, Menlo Park, USA; 10 Virginia Commonwealth University, Richmond, USA; 11 The National Institute on Drug Abuse, North Bethesda, USA; 12 University of Tennessee, Knoxville, USA

**Keywords:** Screen media activity, Screen time, Passive sensing, Android, iOS, Adolescents, Smartphone use, Psychology, Human behaviour

## Abstract

Youth screen media activity is a growing concern, though few studies include objective usage data. Through the longitudinal, U.S.-based Adolescent Brain Cognitive Development (ABCD) Study, youth (m_age_ = 14; n = 1415) self-reported their typical smartphone use and passively recorded three weeks of smartphone use via the ABCD-specific Effortless Assessment Research System (EARS) application. Here we describe and validate passively-sensed smartphone keyboard and app use measures, provide code to harmonize measures across operating systems, and describe trends in adolescent smartphone use. Keyboard and app-use measures were reliable and positively correlated with one another (*r* = 0.33) and with self-reported use (*r*s = 0.21–0.35). Participants recorded a mean of 5 h of daily smartphone use, which is two more hours than they self-reported. Further, females logged more smartphone use than males. Smartphone use was recorded at all hours, peaking on average from 8 to 10 PM and lowest from 3 to 5 AM. Social media and texting apps comprised nearly half of all use. Data are openly available to approved investigators (https://nda.nih.gov/abcd/). Information herein can inform use of the ABCD dataset to longitudinally study health and neurodevelopmental correlates of adolescent smartphone use.

## Introduction

Youth screen media activity (SMA; see Table 1 for acronyms) is a significant public health concern, though research has identified both positive and negative associations between SMA and health, cognitive, and other outcomes^1–3^. Some have hypothesized that adolescents may be particularly vulnerable to the impact of screen use, positive or negative, due to ongoing neurodevelopment^4,5^. Yet knowledge about the relationship between screen usage and developmental, cognitive, and mental health outcomes is hampered by concerns regarding the measurement of SMA behaviors, which have generally been measured via self-report surveys^6,7^.

**Table 1:** Key terms and acronyms.

Term	Definition
ABCD Study	Adolescent brain cognitive development study, a longitudinal study of 11,878 community-recruited diverse youth in the United States
ABCD-EARS	An ABCD-specific version of EARS sensing keyboard use (Android and iOS) and screen usage (Android only) for the device on which it was installed
Android	Android Operating System
App	Application on a smartphone
App Store	Application store for iOS devices
App Use	Screen display time of each app used on Android devices, as measured by ABCD-EARS
EARS	Effortless Assessment Research System, an application with multiple sensor capabilities designed by Ksana Health, Eugene, OR
Google Playstore	Application store for Android devices
iOS	Apple’s Operating System
Keyboard use	Detection of keyboard activation within an app; indicates app usage and engagement on Android and iOS devices
Keystrokes	Number of keystrokes on Android and iOS devices as measured by ABCD-EARS
NDA	National Institute on Mental Health Data Archive is a data repository for NIH-sponsored research studies; access to ABCD data can be applied for through here
OS	Operating system on a smartphone
SMA	Screen Media Activity, an overarching term for all screen use

While self-report surveys offer a flexible, readily implementable first step in the study of emerging public health phenomena, like excessive smartphone-based SMA, these surveys are subject to many well-known limitations^8^. Self-report surveys are effortful for participants to complete and are thus generally collected at infrequent intervals. They are also limited by participant reporting and recall biases, with prior research indicating that youth often inaccurately estimate their actual SMA on self-report instruments^6,7^. Youth technology use patterns, such as the most popular social media sites or smartphone applications, also change rapidly. Self-report SMA assessments therefore require constant updates to accurately query how screens are being used and frequently include outdated items^6^. Hence, self-report measures of SMA may often be error-prone and poorly harmonized across time.

More accurate measurement of smartphone-based SMA may be possible through remote sensing applications, which passively and objectively measure smartphone application (“app”) use. Measures of SMA based on passive sensor data offer several advantages over typical retrospective self-report-based measures. Namely, passive monitoring methods are not subject to self-report biases, offer flexibility in capturing newly popular categories of SMA, and require minimal participant effort, allowing for frequent periods of continuous assessment^9,10^. While still subject to other sources of error (e.g. devices left on in the background), passive sensing-based methods nonetheless offer a promising tool for improving the accuracy, flexibility, and scale of SMA data collection^11^.

Despite potential benefits of passive sensing, these methods come with their own unique challenges. For example, differences between smartphone operating systems (OS) can limit data comparability or accessibility. The vast majority of studies using smartphone sensor data to measure app usage have been conducted with Android devices, as these have historically allowed third-party applications greater access to sensor data than other devices, such as Apple iOS^12^. However, 87% of teenagers in the United States use Apple smartphones^13^, suggesting our understanding of adolescent smartphone use patterns may be based on non-representative samples. Recently, the Adolescent Brain Cognitive Development (ABCD) Study^14^, a large, longitudinal, and diverse cohort of U.S. adolescents, implemented passive sensing of youth smartphone use via app use monitoring^7^. To better capture information on SMA in both Android and iOS users, a third-party keyboard overlay collected keyboard metadata, such as the application in the foreground during each keystroke. This allowed for the creation of “proxy” measures of smartphone use/app engagement that could be calculated across operating systems, such as the number of keystrokes recorded when using a particular app. While keyboard measures did not capture SMA not involving the keyboard, they nonetheless offer a means of passively sensing SMA in iOS device users, where data collection restrictions preclude the collection of other passive sensor measures of SMA. Importantly, keystrokes are shown as a unique marker for various disease risk states based on metadata, yet accessibility to participants (e.g., by age), methods for analyses, and interpretation of data vary^15–17^. Even so, together, use of keystrokes data expands the reach of remote sensor-based SMA measurement to include the vast majority of U.S. youth who use iOS devices and provides novel information on smartphone use by teens regardless of iOS or Android operating systems.

The present manuscript has three aims. First, we present methods for reconciling SMA usage metrics based on two passively sensed summary measures (average daily app use and keystrokes) and describe a method for cleaning and using this open science dataset in Android and iOS users. Second, we address the measurement properties of passive sensor measures of keyboard and app use and describe the initial validation of these two summary measures of youth smartphone-based SMA within a large, diverse sample of U.S. adolescents. Third, we detail observed trends in youth smartphone use, including intra-day patterns of smartphone use, the quantity of smartphone use recorded across different app categories, and differences in patterns of smartphone use by participant sex and device OS, and share code for conducting similar analyses. Based on prior findings^7^, we expected moderate correlations (e.g., r = 0.4-0.59) between app use, keyboard, and self-report measures of youth smartphone use. We further expected that self-report measures would underestimate smartphone use relative to passively monitored app use. Lastly, we predicted that passive keyboard measures would correspond better with app usage for apps requiring more active engagement (e.g., messaging, social media) than for more passive apps (e.g., video streaming, reading).

## Methods

### Participants

The ABCD Study is a 21-site 10-year longitudinal study with 11,880 participants at baseline (ages 9–10), funded by the National Institutes of Health^18^. At the 4-Year follow-up, participants and their parents/guardians completed annual visits and all participants were offered the opportunity to participate in an additional aspect of the ABCD protocol to passively measure youth participant smartphone use via the ABCD Effortless Assessment Research System (EARS) app. Here we examine cross-sectional data from ABCD Data Release 5.0 (10.15154/8873-zj65), collected between September 2019 through January 2021, containing the first half of Year 4 follow-up data (total n = 4754; a subset, n = 1,463, had usable ABCD-EARS data). Participant demographics, stratified by participant operating system, are reported in Table 2, along with US demographic characteristics of 10–14-year-olds^19^.

**Table 2:** Participant demographics by operating system (N = 1463).

Demographics	Apple, n (%)	Android, n (%)	ACS^b^ (2021)
n	962	501	
Age M (SD), range	14.2 (0.66) 12.7–15.7	14.0 (0.70) 12.6–15.8	
Sex			
Male	426*^✝^ (44.2%)	292*^✝^ (58.3%)	51.2%
Female	536*^✝^ (55.7%)	208*^✝^ (41.5%)	48.7%
Missing/declined/other	0 (0.0%)	< 10 (< 0.2%) ^c^	
Race/ethnicity			
Asian	23^✝^ (2.4%)	12^✝^ (2.4%)	5.0%
Black	93^✝^ (9.7%)	56 (11.2%)	13.5%
Hispanic	158^✝^ (16.4%)	97^✝^ (19.4%)	25.9%
White	588^✝^ (61.1%)	284^✝^ (56.7%)	52.3%
Other/more than one race	100^✝^ (10.4%)	52^✝^ (10.4%)	27.8%
Annual household income			
< $50,000	167*^✝^ (18.4%)	169* (36.4%)	36.1%
$50,000—$100,000	277 (30.5%)	155 (33.4%)	28.1%
≥ $100,000	465*^✝^ (51.2%)	140* (30.2%)	35.8%
Missing/declined	53 (5.5%)	37 (7.4%)	
Parent educational attainment^a^			
Less than high school diploma	28^✝^ (2.9%)	16^✝^ (3.2%)	8.9%
High school diploma or GED	48^✝^ (5.0%)	36^✝^ (7.2%)	27.8%
Some college	242*^✝^ (25.2%)	159*^✝^ (31.9%)	14.9%
Bachelor’s degree	269^✝^ (28.0%)	145^✝^ (29.1%)	23.5%
Post-graduate degree	375*^✝^ (39.0%)	143*^✝^ (28.7%)	14.4%
Missing/declined	0 (0.0%)	< 10 (< 0.2%)	

*Differs between Apple and iOS users (*p* < *.05*).

^✝^Differs from American Community Survey (p < .05).

^a^Highest education level achieved by either parent.

^b^ACS sex and race/ethnicity values are for 10–14 year old children living in the US, and household income and parent educational attainment are for US adults, both from the 2021 American Community Survey (https://data.census.gov/table/ACSST1Y2022.S0101). ACS race categories sum to > 100% because Hispanic/Latino is recorded as an ethnicity rather than a race.

^c^Cell sizes < 10 are collapsed to prevent identification of participants, consistent with NIH reporting guidelines.

### Procedure

The ABCD Study protocol, including the ABCD-specific Effortless Assessment Research System (EARS; https://ksanahealth.com/ears/^20,21^) app, was approved by the IRB at the University of California San Diego and performed in accordance with the relevant guidelines and policies, including the Declaration of Helsinki. ABCD-EARS Application enrollment procedures were similar to a pilot substudy within the ABCD cohort at the Year 2 follow-up^7^ and used the same ABCD-EARS app on participant’s own smartphones. Full recruitment and study protocol information is available elsewhere^22–27^. Participants and their parents/guardians provided written informed consent for each annual visit and ABCD-EARS participation. Whether in-person or remote, consented participants with compatible smartphone devices (Android phones with Android OS version 6 or newer and iPhones 7 Plus or newer) downloaded the ABCD-EARS application with the help of trained research assistants, including the accompanying EARS keyboard. Families were compensated for their time, with additional compensation offered for the ABCD-EARS protocol.

After participants enabled data collection each day, whenever a participant entered a keystroke on their smartphone device’s keyboard, the ABCD-EARS application recorded: (1) the active application in the foreground of the participant’s phone, (2) the application’s App Store/Play Store category, (3) the number of keystrokes input (referred to as keystroke data), and (4) the times at which the keyboard was opened and closed. In Android device users, the ABCD-EARS application recorded the times at which a participant opened, closed, or minimized an application from the foreground of their device (referred to as app use). Data were uploaded to a secure, remote server for storage up to every few minutes and at least once a day. Further information on how the ABCD-EARS application data were recorded, cleaned, and processed are available in the online Supplement S1.

### Measures

#### Sociodemographics

Parents reported sociodemographic characteristics during the baseline visit, including youth sex assigned at birth, combined family household income, and parental education^22^. Gender was also reported by the youth using a two-step method, first querying sex assigned at birth then asking their current gender identity (boy, girl, another gender [e.g., nonbinary])^28^; data by gender identity, rather than sex assigned at birth, are included within the Supplement S1. As sociodemographic characteristics include social constructs that require careful interpretation in light of appropriate contextualizing factors^29^, sociodemographic factors are included for description but limited in interpretation.

#### Screen time questionnaire

All participants in the ABCD Study completed annual surveys on their SMA. This included screen-based activities across all devices, excluding time spent on schoolwork. Beginning at Year 4 follow-up, participants separately reported overall SMA and, if they had their own smartphone, screen time on their smartphone. Typical weekday (Monday-Friday) and weekend (Saturday/Sunday) use were self-reported by youth for overall usage and usage by device type (e.g., tv vs. smartphone) and specific type of media (i.e., streaming TV/movies, playing single-player video games, playing multiplayer video games, texting, on social media, using video chat, and total time). Only items related to smartphone usage were included in the present analyses.

### ABCD-EARS-derived measures

#### Average daily keystrokes and app use

The ABCD-EARS application recorded the time of each keystroke made on the ABCD-EARS keyboard (iOS) or the device’s native keyboard (Android) as well as the application open in the phone’s foreground at that time. To ensure participant privacy, the application did not record keyboard use content (e.g., what participants were typing). The Android version of the application also passively collected app usage data. Average number of keystrokes recorded and minutes of app use by a participant per day by app category and overall are reported. Further information on data collection and measurement definitions are available in the Supplement S1.

#### App category harmonization

Application categories are labeled by app creators/publishers when uploading to the Google Play Store and Apple App Store and therefore varied by operating system. Accordingly, data from Android phones only contain categories from the Google Play Store, whereas data from iOS phones only contain categories from the Apple App Store. In order to have comparable keyboard data across operating systems, the ABCD Novel Technologies Workgroup devised a method to integrate and harmonize data regardless of OS (see Table 3 and Supplement S1).

**Table 3:** Data harmonization of Apple iOS and Android OS categories for ABCD-EARS keyboard measures.

ABCD category	Apple iOS categories	Android OS categories	Self-report category
Books	Book + reference	Books and reference	
Business	Business	Business	
Education	Education	Education	
Entertainment	Entertainment	Comics + entertainment + events	Streaming Movies/TV
Finance	Finance	Finance	
Food	Food and drink	Food and drink	
Games	Games + kids	Action + adventure + arcade + board + card + casino + casual + educational + music + puzzle + racing + role playing + simulation + sports + strategy + trivia + word	Single player gaming + multiplayer gaming
Art	Graphics and design	Art and design	
Health	Health and fitness	Health and fitness	
Lifestyle	Lifestyle	Beauty + house and home + lifestyle + parenting	
Music	Music	Music and audio	
Navigation	Navigation	Maps and navigation	
News	Magazines and newspapers + news	News and magazines	
Medical	Medical	Medical	
Photography	Photo and video	Photography + video players	
Productivity	Productivity	Productivity	
Shopping	Shopping	Autos and vehicles + shopping	
Social	Social networking	Communications + dating + social	Texting + video chatting + social media
Sports	Sports	Sports	
Travel	Travel	Travel and local	
Tools/utilities	Utilities + developer tools	Libraries and demo + personalization + tools	
Weather	Weather	Weather	

### Analyses

All analyses were run in RStudio using R version 4.2.3^30^.

#### Reliability and variability of smartphone sensing measures

To quantify the inter-day variability and internal consistency of keystroke and app usage measures, we computed both intraclass correlation coefficients (ICCs; single random raters) and Cronbach’s *αs* for daily app use and daily keyboard use. We first computed total keystrokes and minutes of app use for each day of study participation. ICCs and *αs* were then obtained for daily keystrokes and passively measured smartphone use using the ‘psych’ package^31^. Due to data collection demands for a concurrent study on wearable sensor data, 15.9% of the study sample provided more than the intended 21 days of data, though only their first 21 days of data were included in these analyses.

#### Validation of summary smartphone measures

To ensure that keystroke and app usage measures were capturing usage patterns consistent with typical sleep/wake cycles, we computed the average amount of screen time captured by time of day from the ABCD-EARS daily keyboard and app use data. To do so, we binned observations by time of day and computed the average keystrokes and minutes of app use, inferring that valid keystroke and app use measures ought to offer concurring accounts of intra-day use patterns and further show that smartphone use is lowest late at night, when participants are expected to be asleep. Code to calculate hourly smartphone use from the ABCD-EARS data is available on OSF (https://tinyurl.com/3hutza88).

Next, to determine whether average daily keystrokes, average daily app use, and self-reported smartphone use offered consistent accounts of participant screen time, we conducted several tests of alternate-forms validity (e.g., the extent to which different measures of the same construct are consistent) for these measures. We first computed the mean proportion of daily keystrokes, minutes of daily app use, and minutes of self-reported smartphone use that were recorded in each application category. We then fit models for the relationship between proportion of application use across screen time measures via a series of beta regressions, replacing proportions of 1 with 0.999 and 0 with 0.001 to ensure all values meet the requirements of the beta regression (Cribari-Neto & Zeileis, 2010). We inferred that greater correspondence in the proportion of application use across measures would offer stronger evidence for their validity.

To further test the degree to which the three smartphone use measures offered concurring accounts of smartphone application use, we computed Pearson correlation coefficients between average daily keystrokes, average daily app use, and self-reported smartphone use, inferring that greater correlations between measures would show greater evidence for the convergent validity of each measure. As the ABCD-EARS application differed between iOS and Android devices, this analysis was stratified by participant operating system. To test whether the correspondence between the three screen time measures varied between different smartphone use activities (such as between video streaming and social media use applications), we stratified these analyses according to application category. Lastly, we fit a Bland–Altman plot of average daily app use and self-reported screen time in android users to visualize the degree of correspondence between these measures across different levels of self-reported and passively recorded smartphone use^32,33^.

#### Smartphone use by sex and operating system

After investigating whether ABCD-EARS summary variables were reliable and offered valid measures of participant smartphone use, we next investigated associations between smartphone use and participant characteristics: namely, sex and smartphone operating system. Two-sample *t*-tests were used to test whether summary measures of screen time differed as a function of participant sex or operating system with statistical significance assessed parametrically and again via bootstrapped 95% confidence intervals (k = 4000 replications; see Supplement S1). Bootstrapping was used as data are skewed and we wanted to ensure the robustness of the results. We did not use a multivariate approach as we were assessing for direct group differences rather than group differences while controlling for covariates.

#### Missing data

Remote sensor data often have substantial missing data (Hicks et al., 2019). Furthermore, in the case of smartphone data, it can be challenging to determine whether missing data arises due to validly recorded periods of low screen use or due to data recording lapses. When defining data as “missing,” we applied a conservative definition of “missing” data, as a full day on which no data was recorded on a particular measure. This was done to reduce the likelihood that periods of non-screen usage would be falsely categorized as missing data. We then computed the proportion of missing data for each measure as the proportion of “missing” days during the first 21 days of data collection. To assess the degree to which smartphone measures and participant demographics were associated with missing data, we computed associations (Pearson correlations) between the proportion of missing days of keyboard and app use data with participant operating system, demographics, and smartphone measures.

## Results

### Descriptive statistics of self-reported and passively-sensed summary measures

#### Differences between youth who with passive sensing and those without

Self-reported smartphone use was not significantly different between ABCD study participants who temporarily installed EARS on their device than amongst those who did not (t_2839_ = 0.86, *p* = 0.06; EARS-yes mean = 184.64 min, SD = 161.90; EARS-no mean = 174.80 min, SD = 159.93). Only 16% (n = 514) of youth who declined to participate in EARS did *not* have a smartphone. Participants who participated in EARS did not differ by household income, but did significantly differ by age, sex, parental education, and race/ethnicity (*p*’s < 0.05; see Table S2).

#### Descriptives of youth who participated in passive sensing

On average, youth with EARS self-reported 62% less screen time per day than was estimated via passively sensed average daily app use (185 ± 162 vs 298 ± 174 min; t_474_ = 15.5, *p* < 0.001). EARS recorded 1,221 ± 1,884 keystrokes per day on average. The distributions of self-reported use, average daily app use, and average daily keystrokes were all right-skewed (see Supplemental Figs. S1–S3); a small number of outlying participants recording far higher average daily keystrokes and self-reported smartphone usage than the rest of the sample. Neither average daily keystrokes nor average daily app use differed significantly between weekends and weekdays (*t*s = 1.08–1.45, *p*s = 0.15–0.28), nor did their correlations with self-reported weekend/weekday smartphone use (app use and self-report: r = 0.29 on weekends; r = 0.32 on weekdays; keystrokes and self-report: r = 0.09 on weekends; r = 0.11 on weekdays). Therefore, overall daily averages are reported. See additional descriptive statistics in Supplemental Table S3.

### Variability of screen time measures

On average, within-subject variability in participant daily keystrokes, as measured by their between-day standard deviation, was 871.8 keystrokes per day while variability in daily app use, again measured by between-day standard deviation, was 2.4 h per day. Intraclass correlation coefficients (ICCs) for daily keystroke count and minutes of app use were 0.58 and 0.51 respectively, indicating both measures were reasonably consistent within participants, though with considerable inter-day variability in individual screen use (see Supplementary Table S4). Cronbach’s *α*s for daily minutes of app use (α = 0.96) and daily keystroke count (α = 0.98) indicated split-half reliabilities for days in the study of > 0.95, suggesting that, despite individual daily variability, average levels of both participant app use and keystrokes per day were highly internally consistent.

### Smartphone use by time of day

**Figure 1:**
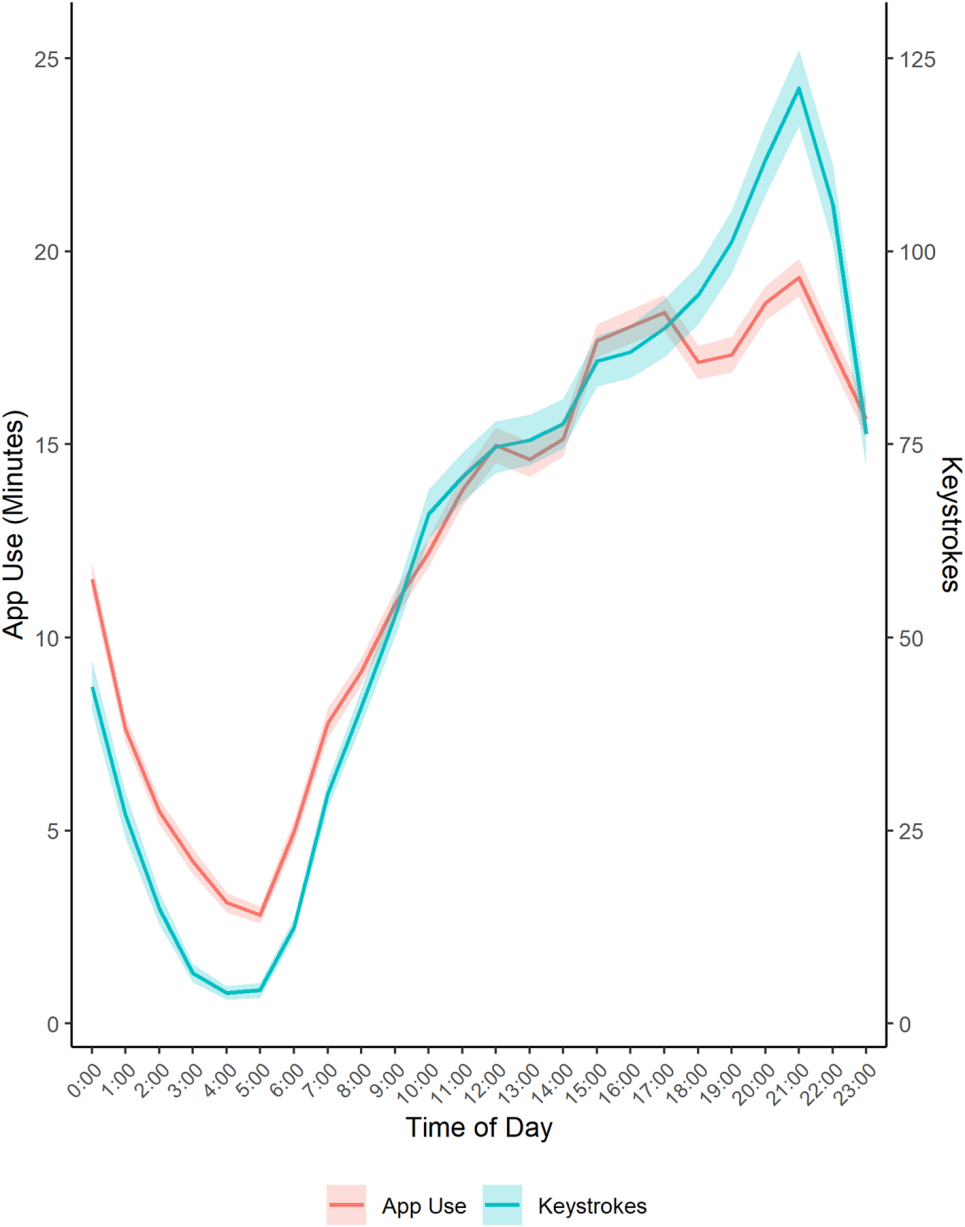
Average smartphone use (app use and keystrokes) by time of day. Passively sensed keystrokes and app use by hour of the day across the full duration of the study. Shaded bands represent 95% confidence intervals. Passively sensed app use was only captured for participants with Android smartphones while passively sensed keystrokes include both Apple and Android participants.

To investigate patterns of youth smartphone use by time of day and whether keystroke and app use measures reflected intra-day screen time patterns, we plotted the average amount of screen and keyboard use recorded during each hour of the day (see Fig. 1). Keystroke and app use measures provided similar accounts. Use was lowest between 2 and 5 AM and increased gradually throughout the afternoon and evening, peaking between 8 and 10 PM. On average, Android participants recorded 7.8 min/hour screen time at all hours of the day. Between 12 and 5 AM on week nights, 47% of participants recorded at least one keystroke per night and 80% of Android users passively recorded at least one minute of app use per night.

### Smartphone use by application category

**Figure 2:**
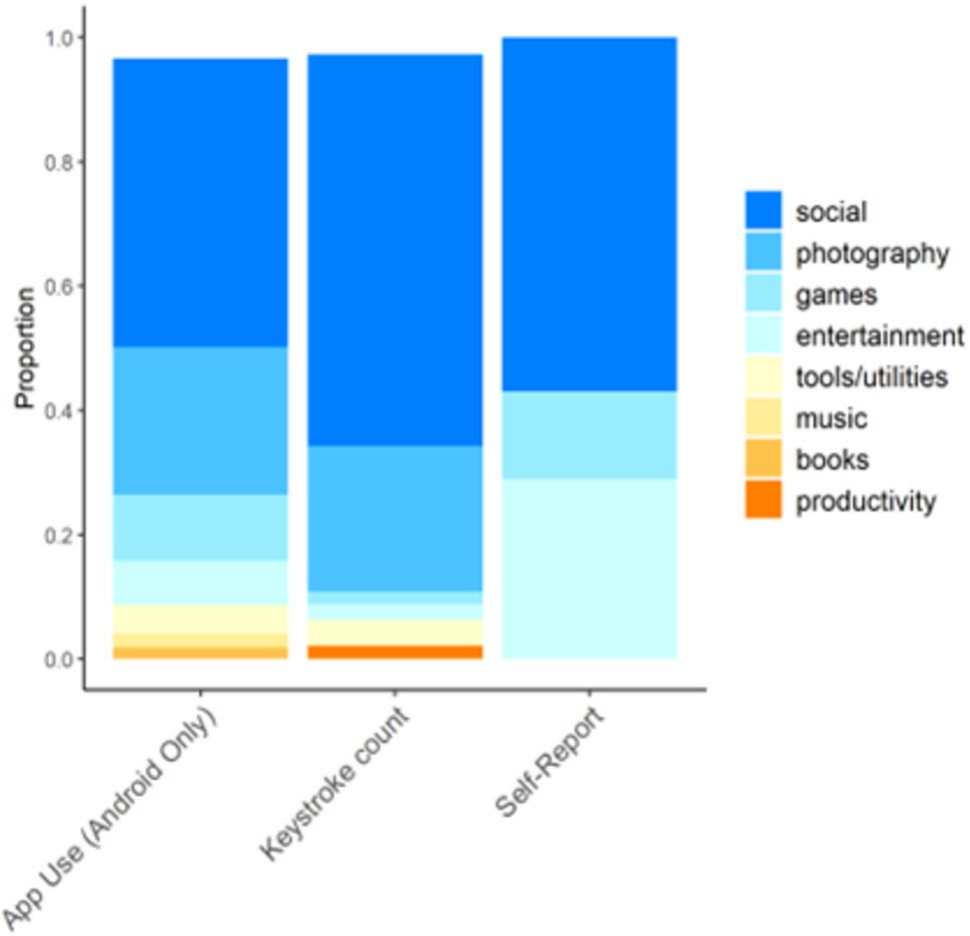
Proportion of app use by category across data sources. Proportion of daily overall application use by category, as measured by average daily app use, average daily keystrokes, and self-reported smartphone use on the Screen Time Questionnaire for their own usage of each category. Only categories representing more than 1% of daily average use are plotted (categories representing less than 1% of daily smartphone use include “Business”, “Education”, “Finance”, “Food”, “Art”, “Health”, “Lifestyle”, “Maps”, “News”, “Medical”, “Shopping”, “Sports”, “Travel”, and “Weather”).

The ABCD-EARS keyboard recorded a mean of 1.44 (SD = 1.15; range = 1–18) unique applications used per day across both iOS and Android users. Among Android participants, passively sensed app use recorded significantly more unique apps used per day than the ABCD-EARS keyboard (2.87 vs. 1.28; *t*_*476*_ = 30.2, p < 0.001), as some apps require no keyboard engagement. Despite differences in apps used per day recorded across measures, keystroke and app use measures exhibited substantial agreement on the mean *proportion* of smartphone usage recorded in each application category (see Fig. 2; see Supplementary Table S1 for example apps by category). For Android users, average daily keystrokes recorded a higher proportion of “Social” applications (β = 0.205, p < 0.001) and average daily app use recorded a higher proportion of “Photography,” “Games,” and “Entertainment” applications (β = 0.237–0.866, *p*s < 0.001).

Participants self-reported spending the highest proportion of smartphone use on streaming, followed by social media use, texting, and video chatting (see Supplement S1 for details).

### Correlations between smartphone measures

Among Android-users, average daily keystrokes and average daily app usage were modestly correlated (*r*_475_ = 0.33, *p* < 0.001); self-reported smartphone use modestly correlated with average daily app use (*r*_473_ = 0.35, *p* < 0.001). For iOS and Android participants, self-reported smartphone use was modestly correlated with average daily keystrokes (*r*s_461-1395_ = 0.13–0.21, *ps* < 0.001; see Supplementary Tables S5 and S6).

A Bland–Altman plot displaying the relationship between the mean of average daily app use and self-reported smartphone use and their difference is presented in Supplementary Fig. S4. Android users recorded mean = 136 min more average daily smartphone use than self-reported, though there was substantial individual variability in this difference (SD_Dif_ = 191.7 min), with variability increasing with greater reported/recorded smartphone use.

### Differences in application usage by sex and operating system

**Figure 3:**
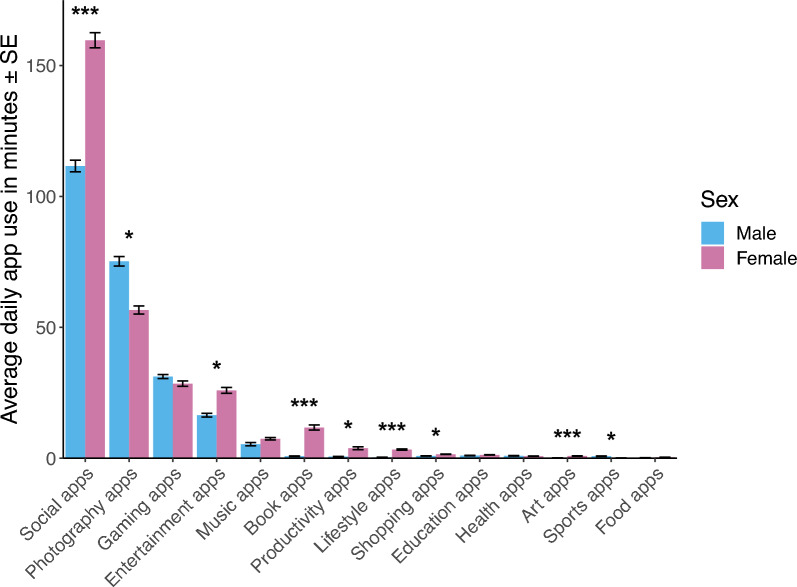
Average daily app use for by female (n = 208) and male (n = 292) for Android using participants. *Sex difference at *p* < 0.05 *** sex difference at *p* < 0.001.

Female participants recorded significantly more average daily app use (*t*_1252.3_ = 6.9, *p* < 0.001; see Fig. 3 and Supplement S1), average daily keystrokes (*t*_428.9_ = 3.57, *p* < 0.001), and self-reported smartphone use (*t*_1404.5_ = 8.93, *p* < 0.001) than male participants.

Average Daily Keystrokes by category and operating system are presented in Table 4 and Supplement S1. Android users recorded more daily average keystrokes than iOS users on average (t_806_ = 3.4, *p* < 0.001).

**Table 4:** Average daily keystrokes by app category and operating system.

Category	Android users, mean (SD)	iOS users, mean (SD)
**Social**	1062.20 (1976.47)	519.66 (995.08)
**Photography**	22.86 (53.30)	387.87 (976.75)
**Tools/utilities**	32.17 (43.48)	54.78 (73.98)
Games	38.88 (363.37)	14.23 (71.83)
**Entertainment**	7.29 (23.09)	38.62 (111.23)
Productivity	15.25 (109.01)	28.09 (127.43)
Music	8.05 (78.68)	6.15 (17.65)
**News**	0.02 (0.23)	5.83 (70.85)
Lifestyle	5.63 (46.16)	2.29 (14.15)
Books	2.99 (32.54)	4.92 (78.02)
Shopping	4.37 (13.46)	3.77 (24.34)
Business	1.36 (9.60)	0.57 (5.83)
**Travel**	0.70 (4.54)	0.09 (1.02)
**Maps**	0.003 (0.08)	0.56 (2.29)
**Food**	0.49 (2.33)	0.21 (1.39)
Sports	0.27 (2.34)	0.28 (3.09)
Art	0.16 (1.75)	0.11 (2.24)
Medical	0.03 (0.39)	0.13 (3.87)
**Weather**	0.01 (0.12)	0.08 (0.50)

Category	Android users, mean (SD)	iOS users, mean (SD)
Unique apps used per day	1.28 (0.93)	1.52 (1.24)
Days with at least one recorded keyboard use	17.42 (6.98)	10.43 (7.2)

Average keystrokes per day spent using each application category recorded via the ABCD-EARS keyboard data in Apple iOS and Android users. Bolded categories indicate p < .05 difference by operating systems.

### Missing data

On average, during the first 21 days of study participation, participants recorded at least one keyboard session on 13.25 days (SD = 0.35 days) while Android participants further recorded at least one app use observation on 19.5 days (SD = 0.18 days). Android users had significantly fewer missing days of app usage than missing days of keyboard usage (7% days missing vs. 13% days missing; *t*_482_ = -8.1, *p* = 3.9*10^–15^). Furthermore, iOS users had more missing keyboard days than did Android users (49% days missing vs. 13% days missing, t_1250_ = 22.2, *p* < 2 * 10^–16^).

Participant age and household income were both significantly associated with missingness, with older participants recording significantly more days missing keyboard data (β = 0.003, p = 0.002) and participants from households making less than $50,000 dollars per year recording significantly more missing app usage days (β = 0.056, p = 0.033). Sex, race/ethnicity, and parental education were not significantly associated with missingness in either measure.

Missing data was also a significant predictor of smartphone measures themselves. Apple users with more days of missing keyboard data recorded fewer average daily keystrokes (β = − 533.4, *p* < 2 * 10^–16^) but *increased* self-reported screen time (β = 11.1, *p* = 0.04). Android users with more days of missing keyboard data had fewer daily keystrokes, (β = − 245.11, *p* = 0.01) and less average daily app usage (β = − 45.3, *p* = 1.9 * 10^–8^). Finally, Android users who had more missing days of app usage also recorded less average daily app usage overall (β = − 30.5, *p* = 9.5 * 10^–5^).

## Discussion

Passive sensing data is a useful measure of smartphone-based SMA^6^. First, evidence here suggests passive sensor data from the ABCD-EARS application provide novel, informative measures of smartphone use in adolescents that offer important insights into teen smartphone use across operating systems, revealing both consistency between subjects and variability within subjects. Together, preliminary evidence from the pattern of results across analyses suggest passive sensing of app use and keystrokes are valid and reliable for between- and within-subject investigations. Second, findings indicate that teens use their phones significantly more than they report, especially females, with much of that time devoted to social media and texting apps. Third, more temporally specific daily or hourly smartphone use measures can be determined from passive sensing, facilitating examining daily or weekly smartphone use patterns, and present analyses revealed surprising levels of smartphone use even in the middle of the night. The full open-science ABCD dataset contains thousands of variables collected longitudinally across adolescent development (including neuroimaging, cognitive performance, substance use, mental health, physical health, and genetics data). Combining this rich ABCD-EARS data with the full dataset is a remarkable asset for the scientific community to inform our understanding of the impact of smartphone-based SMA, including shaping family rules and policies for teen smartphone use.

The present study investigated convergent validity of two passively sensed measures of smartphone use in adolescent participants: average daily keystrokes and app use. Measures were largely stable within participants, offered concurrent accounts of intra-day use patterns, and identified the same most-used application categories, though with significant differences in the proportion of use attributed to each of these categories. Average daily keystrokes and app use were positively correlated with one another, though modestly, suggesting they capture different features of youth SMA and are susceptible to different sources of error. As expected, correlations between average daily keystrokes and app use were strongest for activities that most required active keyboard use, such as gaming, texting, or interacting with social media apps, and weakest for more passive screen uses, such as streaming video or listening to music. Together this suggests that average daily keystrokes are a reasonably effective measure of smartphone use in apps that require more engagement.

Both average daily keystrokes and app use were positively correlated with self-reported smartphone use, although modestly so. This is consistent with prior findings on the association between passive sensing and self-report^7^ and may reflect error unique to self-report, such as reporting and recall biases^8,10^. Additionally, a Bland–Altman plot revealed substantial variability in the discrepancy between self-reported smartphone use and daily app usage, which increased as participants recorded more smartphone use. Thus, preliminary evidence here suggests that between- and within-subject investigations of keystrokes and app usage may be captured passively, which may alleviate financial, personnel, and timeliness burdens in studies which capture real-time high-frequency data such as smartphone use. Importantly, there is no gold standard method for measuring SMA yet, whether through self-report or passive sensing, as gold standards require general consensus across the field from a measure which offers known results (including known limitations) and can be reasonably implemented^34^. While concurrent validity and reliability of ABCD-EARS can be further investigated in the future through assessment against other methods (e.g., manually timed measurement of app use or keystrokes), the present findings provide an example of the strengths of incorporating novel technology into real-time monitoring of aspects of smartphone use behavior. Results suggest that self-reported screen time may be estimated imprecisely relative to average daily app use, further highlighting the potential value of objective smartphone use measures.

Interestingly, associations between self-reported smartphone use and average daily keystrokes differed between Apple and Android device users, possibly due to differences in participant characteristics, data collection procedures (e.g., the use of a third-party keyboard in iOS devices), and smartphone use behaviors between Android and iOS users. Even so, these findings provide further evidence for the use of passive sensor measures of smartphone use, though also highlight the need to control for operating system effects in analyses using these variables.

Patterns of missing data differed across sensor measures, operating systems, and participant sociodemographics. Furthermore, the extent of missing data strongly predicted average daily keystrokes and app use as well as self-reported smartphone use. Missing data were particularly prevalent for iOS keystroke data, likely reflecting the fact that the third party ABCD-EARS keyboard was required, which some participants may have found aversive, and which was occasionally automatically replaced with the native iOS keyboard by their phone. Participants may have also disabled the keyboard themselves at times. Although these rates of missingness are not out of line with the field of sensor-based phone data (e.g.,^35^), caution is warranted to consider reasons for missingness and the potential influence on research aims, particularly with iOS variables. Our findings affirm the importance, frequently emphasized in mobile health research, of exploring and appropriately statistically controlling for missing data when using smartphone sensor data^36^.

In addition to describing data quality of ABCD passive smartphone use measures, the present investigation also offers a compelling initial view into how American adolescents interact with their smartphones. First, consistent with recent research on objectively measured smartphone use in adolescent Android users^37^, adolescents recorded an average of *five hours* per day of smartphone application use. Consistent with our hypothesis, this was two hours more than teens estimated via self-report prior to initiating passive sensing. This suggests that adolescents are spending large amounts of time on their phone each day and that they may be unaware of just how much time they are spending doing so. Indeed, considering the time adolescents spend at school and on schoolwork or household chores, our findings suggest that many adolescents are spending the bulk of their leisure (and perhaps even non-leisure) time on their smartphones. Furthermore, this estimate is reduced by missingness and does not include activities that do not require an application in the foreground, such as music streaming, indicating that even this estimate of 5 h per day may underestimate the actual daily extent of youth smartphone use.

Second, many participants remain active on their smartphones late at night, with 80% of ABCD-EARS Android participants recording app use between midnight and 5AM on weeknights. In addition, nearly half of all participants had logged keystrokes during this same time period, suggesting more active engagement with their smartphones, rather than using an app that may be perceived as facilitating sleep (e.g., turning on white noise or soothing music). There is a robust literature regarding SMA and adolescent sleep outcomes^38^, though directionality has not been firmly established (i.e., whether disrupted sleep leads teens to use their phones at night as they are already awake). The granular level of daily ABCD-EARS data can be combined with the larger ABCD dataset to investigate the correlates of smartphone use, sleep disruption, and neurodevelopmental, mental health, or numerous other outcomes. Shared hourly binning code and access to the ABCD dataset through the NIMH Data Archive can be used by researchers interested in pursuing these questions.

Lastly, “Social” applications, such as social media apps, texting, and video chatting, were, by a wide margin, the most popular smartphone activities for participating adolescents. Smartphones increasingly mediate large parts of adolescent social life, offering adolescents opportunities for fun, social connection, and identity exploration^39^, though with possible risks of cyberbullying, poor self-esteem, and internalizing psychopathology^40,41^. Such concerns appear especially salient for female adolescents, who have higher rates of anxiety, depression, and body image concerns than adolescent boys^42,43^ and, in the ABCD-EARS sample, were also more avid users of social applications. Research has thus far yielded mixed evidence for smartphone-related effects on mental health^40,44^. Nonetheless, this finding highlights the importance of social media in adolescent social life, the need for continued research on its effects on adolescent mental health, and suggests that adolescent girls may be especially at risk for consequences associated with social media use due to increased use. As the ABCD Study plans to continue collection of passive smartphone data at annual visits for at least the next five years, such investigations will be well facilitated by the longitudinal ABCD dataset and ABCD-EARS data in particular.

Potential targets for future investigation and intervention are numerous based on the data derived thus far. Parents may wish to limit their teen’s phone access particularly overnight, given the prevalence of smartphone use at that time. Teens themselves may benefit from feedback on how much they are using their devices, given underreporting which may indicate a lack of self-awareness of their use, though this needs to be tested experimentally. Future data analyses can detail specific apps and their psychiatric correlates, particularly given ongoing public policy debates in the United States regarding these important topics. Such investigations should also consider intersectionality characteristics, as prior research suggests some youth (e.g., LGBTQ youth;^45^) may uniquely benefit from social media given the potential to build community on such apps.

As detailed here, passive sensing includes many possible avenues for improving data collection of difficult to measure constructs, though it also has drawbacks which must be considered in data interpretation^11,46,47^. Accordingly, the results of the present study should be interpreted given the presence of several important study limitations.

First, participants in the study reflect only a portion of the ABCD cohort, with participation in the EARS passive sensing trial substantially associated with participant demographics. Despite comprehensive efforts to maintain confidentiality and collect minimal, but still meaningful, data, privacy concerns may have contributed to a large proportion of ABCD participants declining to participate in data collection. Privacy concerns represent an especially important barriers to recruiting representative samples to remote sensing studies, highlighting the need to engage thoughtfully with participant concerns over what data are collected, how they are used, and the procedures used to secure them. Further, technological issues, such as having an incompatible smartphone or lack of memory space for the ABCD-EARS app, may also have restricted participation in passive sensing despite youth being willing. However, data on these issues are not included in ABCD data releases, and their influence cannot be assessed.

Second, in addition to apparent sampling biases, the study was subject to substantial missing data. While missing days of use may sometimes reflect participants refraining from using their smartphones, they may also arise for artefactual reasons, such as technical issues with the application, device updates, or due to participants deleting or disabling the EARS application. The latter concern may have especially affected Apple iOS device users, who occasionally reported disabling the EARS keyboard, which replaced the native iOS keyboard and was required for keystroke data collection in iOS devices, due to finding it cumbersome to use.

Third, though designed to be passive, participants may still modify behavior due to the presence of the app on their phone. This may arise due to awareness that their use is being monitored, burdens associated with recording (e.g. the EARS keyboard in iOS users), or due to increased battery drainage from the application. Each of these may have affected participant use patterns and impacted the study’s external validity.

Fourth, while the study was racially, ethnically, and socioeconomically diverse, White participants and participants with highly educated parents were overrepresented. While this is consistent with national data on smartphone access [Pew Research 48], it also may be a source of sampling biases. While we observed differences in smartphone use patterns between male and female participants, and included initial exploratory descriptive data by gender identity within the supplement S1; the full dimensionality of gender identity and relationships with SMA should be assessed in future analyses^28,49^.

Fifth, self-reported smartphone use was designed prior to the ABCD-EARS protocol, included different application categories than the ABCD-EARS measures, and did not include time spent on school-related activities, likely deflating associations between self-report and passive sensing measures.

Sixth, several factors impacted the comparability of Android and iOS results. Smartphone applications are sometimes categorized differently by app creators on Apple’s App Store and Android’s Play Store, which may increase error in some categories. While we harmonized app categories to be analogous across iOS and Android devices, due to the number of applications available in each store, it was not possible to ensure that all applications were included in the same categories across operating systems. Thus, some cross-operating system differences in app categories likely reflect measurement error induced by differently categorized apps. Depending on the research question posed, data users may consider independently analyzing Android and iOS categories rather than always harmonizing across operating systems, or calculating summary variables of specific apps using the more granular data available through the NIMH Data Archive.

Lastly, limitations on third-party app data collection in Apple devices prevented the ABCD-EARS app from collecting app usage data from Apple device users. Results from Android participants suggest average daily keystrokes and app use measures offer reasonably concurrent information on youth smartphone use. However, Android and iOS participants differed in their sociodemographics, patterns of missing data, and patterns of association with self-reported smartphone use, suggesting that Android results may not perfectly generalize to iOS users. Notably, beginning in the Year 7 follow-up visit, the ABCD Study and the ABCD-EARS app are working directly with Apple to collect passive app use data similar to the data available for Android users.

Taken together, the ABCD-EARS app provides smartphone use measures that are internally reliable within participants and offer broadly concurrent accounts of when and how teens use their smartphones. This includes their most used app categories and detailed logs of time of use. Smartphone summary measures are moderately intercorrelated, although more robust correlations exist for applications requiring active keyboard use than for more passive activities. ABCD-EARS data have yielded important insights into adolescent smartphone use: typical adolescents spend many hours each day on their smartphones, frequently using them late into the night. Much of this time is dedicated to social activities like texting, video chatting, and using social media apps, highlighting the important role smartphones play in adolescents’ social lives and the importance of continued research into how these digital spaces affect adolescent mental health.

Overall, the present study demonstrates that passive sensing smartphone data can be used to construct measures of estimated smartphone use, with improved richness, accuracy, and flexibility relative to typical self-report measures. Especially when paired with a diverse, large, longitudinal research sample as in the full ABCD Study, these measures offer a powerful tool for improving our understanding of adolescent digital life and its impacts on development.

### Supplementary Information

Supplementary Information.

## Data Availability

Data is available for download to researchers who request and receive approval via the NIMH Data Archive (https://nda.nih.gov/study.html?id=2147). Data is derived from ABCD Study Release 5.0 (10.15154/8873-zj65). Custom code for category harmonization and hourly binning is available on the Open Science Foundation (OSF) and GitHub (https://tinyurl.com/3hutza88).
