# Supplementary material for: Passively sensing smartphone use in teens with rates of use by sex and across operating systems
Source: Sci Rep. 2024 Aug 3;14:17982. doi: 10.1038/s41598-024-68467-8 (PMC11297944; doi:10.1038/s41598-024-68467-8)
Supplement: Supplementary file 1 — Supplementary Information. [file 41598_2024_68467_MOESM1_ESM.docx]

**ABCD-EARS Application Data Collection, Cleaning, and Processing**

**Average Daily Keystrokes*.*** The ABCD-EARS application recorded the time of each keystroke made on the ABCD-EARS keyboard (iOS) or the device’s native keyboard (Android) as well as the application open in the phone’s foreground at that time. This was used to compute the start and end times, foreground application, and number of keystrokes recorded during each keyboard use session. To ensure participant privacy, the application did not record keyboard use content (e.g., what participants were typing). These keyboard data were used to compute a summary measure of participant keyboard usage, referred to hereafter as “average daily keystrokes.” This was defined as the average number of keystrokes recorded by a given participant each day during their participation in the study. In addition to average daily keystrokes across all days during the study, we also computed average daily keystrokes separately for weekday and weekend use (e.g., the average daily keystrokes recorded by a participant on all weekend days during their participation), and for each separate smartphone application category (e.g., the average daily keystrokes recorded by a participant in the “Games” category during their participation).

**Average Daily App Use*.*** The Android version of the application also passively collected app usage data. Every fifteen minutes, the ABCD-EARS app queried the Android UsageStats API to obtain data on the apps a participant had open in the foreground in the previous 24 hours, including timestamps corresponding to the times at which an app was opened in the foreground and when that app was no longer active in the foreground (for example, when the app was closed or minimized or when a participant shut off their phone screen). Similar to the keystrokes data, the app use data was used to compute a summary measure of participant app usage. This measure, “average daily app use,” was defined as the average number of minutes of app usage recorded per day by a given participant during the study. Consistent with average daily keystrokes, in addition to computing average daily app use across all study days, we also calculated average daily app use separately for weekend and weekday use and for each separate application category.

**Keyboard**

**Data collection.** For the three-week duration of the ABCD-EARS protocol, ABCD-EARS ran continuously in the background of the participant’s smartphone. To prevent the app from being closed by the smartphone operating system due to memory and/or storage limits and to enable automatic data upload, the app requested that ABCD-EARS participants open the application once per day. This was facilitated through daily push notifications encouraging participants to open the application. If data did not import from a participant’s device for multiple days, an ABCD staff member contacted the participant to troubleshoot, including ensuring that the ABCD-EARS app was installed correctly, running in the background of their device, and being opened daily for recording of smartphone use data.

Whenever a participant opened the keyboard, a timestamp was recorded for each keystroke, along with the identifier for the application hosting the text box. The release of iOS 16 prevented the application identifier from being collected, so only data collected before this release included the application identifier for iOS users.

**Daily features.** Keystroke data were aggregated into daily metrics with the following steps. First, data were aggregated into sessions based on application (when available) and temporal proximity. A session was defined as a sequence of keystrokes with the same application, with each consecutive keystroke being no more than 60 seconds apart. The length of each session was calculated as the difference between the first and last timestamp of the derived session. Finally, daily metrics were aggregated by counting all keystrokes and sessions per day and summing all session durations for each day. Sessions were assigned to the date of the final keystroke in each session.

**Summary metrics.** Data were aggregated further to derive metrics spanning the entire duration of the study. The category for each application that appeared in the keyboard data was scraped from the App Store (iOS) and the Google Play Store (Android), when available. Then, for each participant, all keyboard session data were aggregated to calculate number of keystrokes, sessions, and keyboard usage duration across two dimensions: day of week (weekday, weekend, all days) and application category. In addition, the total number of unique applications used was totaled across the entire study. Finally, average daily usage metrics were calculated by dividing the above metrics by the number of weekdays, weekend days, and total days that the participant used the EARS application. The period of EARS application usage was defined as the total number of days with at least one keyboard session assigned to that day.

**Additional ABCD-EARS Data and Measures*.*** While not included in the present analyses, additional ABCD-EARS-derived measures of keyboard and app usage, including the duration of each keyboard session in minutes and the number of keyboard or app usage sessions per day are available for download via ABCD Study Release 5.0 on the NIMH Data Archive (<https://nda.nih.gov/study.html?id=2147>). More granular ABCD-EARS data used to construct these screen time summary measures are also available via this same source and include the time of day, duration, and specific smartphone application used during each keyboard or app usage instance.

**App usage**

**Data collection.** Every 15 minutes, EARS queried the Android UsageStats API, which returns aggregated information about the last week of application usage. For each application used, the API returned the duration each application was in the foreground, binned into approximately 24-hour windows, with start and end times arbitrarily defined by the API. The final window always ended at the time of the query, and started at some point in the last 24 hours, such that the final windows of successive queries could be subtracted from each other to deduce the amount of application usage between two consecutive queries (i.e. application usage during that 15-minute window). This accumulation would continue for approximately 24 hours until the start time of the query window reset, and then the accumulation would begin again.

**Data cleaning.** Before the raw data could be subtracted to derive application foreground durations during smaller windows (“non-cumulative” durations), data cleaning needed to occur to ensure that all assumptions needed for subtraction were met.

First, duplicate historical windows, which were expected to be collected due to the repeated one-week queries, were discarded.

Next, the consecutive usage windows that remained needed to have the same start time, until the accumulation reset for the next day. However, the API sometimes generated multiple records offset by several seconds, creating overlapping windows with duplicate information, structured such that they could not be subtracted from one another. The distribution of start times was thus explored to reduce the dataset to a set of usage windows where all consecutive start times were either the same or at least 60 seconds apart.

Finally, non-cumulative foreground durations were derived by subtracting consecutive foreground durations for a given application and usage window start time. Although logically this method should never return negative values, the API occasionally returned information that resulted in negative values for non-cumulative durations. Without access to how the API calculates these inconsistent values, we cannot deduce why this happens; however, in order to only keep sensical data, negative values were recoded as zeros.

**Daily features.** To create daily metrics, non-cumulative foreground durations were summed for each day, for each application. Usage windows were assigned to the usage window’s end date. Daily durations greater than 20 hours were removed, as it was discovered that certain system applications, for example home screen tools, resulted in inflated usage (often around 24 hours) that do not represent actual participant usage.

**Summary metrics.** Data were aggregated further to derive metrics spanning the entire duration of the study. The category for each application that appeared in the application usage data was scraped from the Google Play Store, when available. Then, for each participant, all non-cumulative foreground durations were aggregated to calculate durations across two dimensions: day of week (weekday, weekend, all days) and application category. In addition, the total number of unique applications used was totaled across the entire study. Finally, average daily usage metrics were calculated by dividing the above metrics by the number of weekdays, weekend days, and total days that the participant used the EARS application. The period of EARS application usage was defined as the total number of days with at least one application foreground duration assigned to that day.

Some applications, such as home screen managers and other system applications, result in inflated foreground times per day (often around 24 hours). A list of applications to exclude was derived based on quality assessment of the data. An estimate of screen time excluding these applications was derived for each participant. These applications were not excluded from category-specific metrics, but most of them did not fall into categories of interest. Below is a list of the application identifiers excluded:

• com.android.systemui

• com.lge.qmemoplus

• com.google.android.gms

• com.sec.android.app.launcher

• com.sec.android.mimage.photoretouching

• us.ozteam.bigfoot

• com.pixel.art.coloring.color.number

• com.robtopx.geometryjumplite

• com.google.android.inputmethod.latin

• com.lge.clock

• com.pt.bark

• com.tct.launcher

• com.lge.launcher3

• royaln.Removeunwantedcontent

• com.samsung.android.contacts

• com.google.android.packageinstaller

• com.wssyncmldm

**App Category Harmonization.**

While iOS had 25 application categories at the time of data collection, Android had 47. Thus, the Novel Technology Workgroup recommends collapsing Google categories to align with Apple categories to create new, comparable summaries, with a few exceptions. Category harmonization is described in Table 3, and an R script to combine variables in this way is available on the Open Science Foundation (OSF) and GitHub (https://tinyurl.com/3hutza88); example apps in each category are included in Supplemental Table S1. To calculate the number of participants who used apps within each category, it is recommended that data is filtered within category to those participants who have data >0.

App summary categories also differed between ABCD-EARS measures and self-reported smartphone-based SMA on the Screen Time Questionnaire. Thus, when making comparisons between application use measured by ABCD-EARS data and via self-report, self-report response categories were also collapsed to harmonize them with ABCD-EARS application categories. Self-reported streaming corresponded to the “Entertainment” ABCD-EARS category. ABCD-EARS “Games” was computed as the sum of self-reported time spent on single player and multiplayer video games. Lastly, ABCD-EARS “Social” app use was measured as the sum of self-reported time spent texting, video chatting, and using social media sites.

**Missing Data.** Remote sensor data often have substantial missing data [30, 31]. In the case of smartphones, it can be challenging to determine whether missing data arises due to validly recorded periods of low screen use or due to data recording lapses. When defining data as “missing,” we applied a conservative definition of a full day on which no data was recorded on a particular measure. This was done to reduce the likelihood that periods of non-screen usage would be falsely categorized as missing data. We then measured the proportion of missing data for each measure as the proportion of “missing” days during the first 21 days of data collection. To assess the degree to which missing data affected smartphone use measurement, we calculated associations between the proportion of missing days of keyboard and app use data with participant operating system, demographics, and smartphone use measures.

**Results**

**Self-reported Smartphone Use by Application Category.**

Youth self-reported a significantly lower proportion of “Social” application use (β=-0.601 to -0.458, *ps*<.001) and a higher proportion of “Entertainment” application use (β=0.752-1.001, *ps*<.001) than passively sensed measures. Participants self-reported less use of “Games” apps than was recorded via average daily app use (β=-0.450, *p*<.001) yet more than was captured via average daily keystrokes (β=0.381, *p*<.001), likely due variability across games in requirement for keyboard inputs.

**Correlations between Smartphone Measures.** To examine consistency of average daily app use, keystrokes, and self-reported smartphone use across smartphone activities, we stratified correlations by application categories. In Android users, considering only categories representing >1% of daily smartphone use, daily keystrokes and passively measured app use were highly correlated (*r* >0.5) for “Social” and “Games” apps, moderately correlated (*r*>0.3) for “Photography” apps, and modestly correlated (r>0.1, *p*s<0.001) for “Entertainment,” “Productivity,” “Tools/Utilities,” “Books,” and “Music” categories. Among Android users, self-reported smartphone use was most highly correlated with both average daily app use (*r=0.*48, *p*<0.001) and average daily keystrokes (*r*=0.25, *p*<0.001) for “Social” applications. Among Apple iOS users, correlations between self-reported smartphone use and average daily keystrokes were highest for the “Entertainment” category (*r*=0.17, *p*<0.001) (see Supplementary Tables S4 and S5).

**Differences in Application Usage by Sex and Operating System.** Female participants recorded significantly more average daily app use (*t*_1252.3_=6.9, *p*<.001; see Figure 3), average daily keystrokes (*t*_428.9_=3.57, *p*<.001), and self-reported smartphone use (*t*_1404.5_=8.93, *p*<.001) than male participants. Female participants’ average daily app use reflected significantly more use of “Social”, “Entertainment”, “Books”, “Productivity”, “Lifestyle”, “Shopping”, and “Art” applications than males; male participants recorded significantly more use of “Photography” and “Sports” applications (F_1,492_=4.12-19.71; *p*s<.05; see Supplementary Table S6). Analyses by gender identity revealed individuals identifying as boys demonstrated significantly fewer average daily keyboard strokes and app use than either girls or those who identify as another gender (i.e., nonbinary) (*ps*<.01; see Supplementary Figure S5 for information on app use by gender identity). Those who identified as girls or nonbinary did not differ significantly in any measure

Average Daily Keystrokes by category and operating system are presented in Table 4 and Supplement. Android users recorded more daily average keystrokes than iOS users on average (t_806_=3.4, *p*<.001). Android users recorded more keystrokes in the “Finance”, “Food”, “Social”, and “Travel” categories, and iOS users recorded more keystrokes in the “Entertainment”, “Maps”, “News”, “Photography”, “Weather”, and “Tools/Utilities” categories (*p*s<.05).

To confirm results are not due to non-normality, t-tests were bootstrapped. The bootstrapped 95% confidence intervals (R = 4000) and parametric confidence intervals for sex and operating system differences in keystrokes and app use are consistent (i.e., both the bootstrapped CIs and parametric CIs include 0 or both do not include 0) for 45/48 sex comparisons and 21/23 OS comparisons. In all five cases, the t-tests were non-significant and the bootstraps were significant, indicating none of our significant t-test results reflect type-one errors due to non-normal distributions. For sex comparisons, the t-tests were non-significant while the bootstrapped 95% CIs were significant for app use in the "maps" and "productivity" categories and for keystrokes in the "travel" category. For OS comparisons, the t-tests were non-significant while the bootstrapped 95% CIs were significant for keystrokes in the "business" and "games" categories.

**Table S1.** Example Categories for Each App Category by Operating System

| **Android OS Categories** | **Example App** |
| --- | --- |
| Art and Design | Canva: Design, Photo & Video |
| Autos and Vehicles | Cars.com |
| Beauty | Ulta Beauty |
| Books and Reference | KJV Bible Now |
| Business | Zoom |
| Comics | WEBTOON |
| Communications | WhatsApp Messenger |
| Dating | Bumble: Dating App & Friends |
| Education | Duolingo: language lessons |
| Entertainment | Max: Stream HBO, TV, & Movies |
| Events | Ticketmaster |
| Finance | Cash App |
| Food and Drink | McDonald’s |
| Games (Action + Adventure + Arcade + Board + Card + Casino + Casual + Educational + Music + Puzzle + Racing + Role Playing + Simulation + Sports + Strategy + Trivia + Word) | Monopoly GO! |
| Health and Fitness | Health Tracker |
| House and Home | Zillow |
| Libraries and Demo | Mods Melon Playground Sandbox |
| Lifestyle | Life360: Live Location Sharing |
| Maps and Navigation | Uber – Request a Ride |
| Medical | MyChart |
| Music and Audio | Spotify |
| News and Magazines | NewsBreak |
| Parenting | Findmykids: Location Tracker |
| Personalization | Find My Phone By Clap, Whistle |
| Photography | Photo Maker – GIF Master |
| Productivity | AI Security |
| Shopping | Temu: Shop Like a Billionaire |
| Social | TikTok |
| Sports | MLB Ballpark |
| Tools | QR & Barcode Scanner |
| Travel and Local | Expedia |
| Video Players and Editors | CapCut – Video Editor |
| Weather | Live Weather |

| **Apple iOS Categories** | **Example App** |
| --- | --- |
| Book | Strong's Concordance with KJV |
| Business | Glassdoor \| Jobs & Community |
| Developer Tools | Code - Compile & Run Program |
| Education | ASA's Sailing Challenge |
| Entertainment | Hulu: Watch TV shows & movies |
| Finance | Binance Smart Chain Explorer |
| Food & Drink | Sous Vide °Celsius |
| Games | Water Sort Puzzle: Get Color |
| Graphics & Design | ArtRage Vitae Mobile Painting |
| Health & Fitness | HitMeal・Food & Calorie Tracker |
| Lifestyle | Bumble: Dating & Friends App |
| Magazines and Newspapers | Making Jewellery Magazine |
| Medical | Huckleberry: Baby & Child |
| Music | FM Radio App |
| Navigation | Waze Navigation & Live Traffic |
| News | The Economist |
| Photo & Video | Body Tune - Photo Editor |
| Productivity | Wipr |
| Reference | Merriam-Webster Dictionary |
| Shopping | Poshmark: Buy & Sell Fashion |
| Social Networking | Instagram Feed |
| Sports | Formula 1® |
| Travel | U-Haul |
| Utilities | Remote for TCL Roku TVs |
| Weather | Zoom Earth - Live Weather Map |

Notes: Example apps are top listed free apps from Google Play Store for Android. For Apple, the top “Popular Apps” in the App Store were listed.

**Table S2.** Demographics by Opt-in or Opt-out of Passive Sensing (N = 4,754)

| **Demographics** | **Opt-Out n (%)** | **Opt-In n (%)** |
| --- | --- | --- |
| n | 3,291 | 1,463 |
| Age M (SD) Range | 14.05 (0.68) 12.5-15.7 | 14.15 (0.68)  12.6-15.8 |
| **Sex** |  |  |
| Male | 1770 (53.8%) | 718 (49.1%) |
| Female | 1521 (46.2%) | 744 (50.9%) |
| Missing/Declined/Other | 0 (0.0%) | <10 (<0.2%) ^b^ |
| **Race/Ethnicity** |  |  |
| Asian | 75 (2.3%) | 35 (2.4%) |
| Black | 360 (10.9%) | 149 (10.2%) |
| Hispanic | 724 (21.9%) | 255 (17.4%) |
| White | 1810 (54.9%) | 872 (59.6%) |
| Other/More than one race | 322 (9.8%) | 152 (10.4%) |
| **Annual Household Income** |  |  |
| < $50,000 | 835 (25.4%) | 336 (23.0%) |
| $50,000 - $100,000 | 883 (26.8%) | 417 (28.5%) |
| ≥ $100,000 | 1313 (30.0%) | 620 (42.4%) |
| Missing/Declined | 260 (7.9%) | 90 (6.2%) |
| **Parent Educational Attainment ^a^** |  |  |
| Less than High School Diploma | 147 (4.5%) | 44 (3.0%) |
| High School Diploma or GED | 261 (5.0%) | 84 (5.7%) |
| Some College | 795 (7.9%) | 401 (27.4%) |
| Bachelor’s Degree | 887 (27.0%) | 414 (28.3%) |
| Post-Graduate Degree | 1196 (36.3%) | 518 (35.4%) |
| Missing/Declined | <10 (<0.2%) | <10 (<0.2%) ^b^ |

**^a^** highest education level achieved by either parent.

^b^ Cell sizes <10 are collapsed to prevent identification of participants, consistent with NIH reporting guidelines.

**Table S3. Overall Smartphone Use by Self-Report and Passive Sensing**

| Category | Passively Sensed Minutes | Self-Reported Minutes | t and p-values |
| --- | --- | --- | --- |
|  | Mean (SD)  *Median [IQR]* | Mean (SD)  *Median [IQR]* |  |
| Daily Average (Total) | 293.3 (172.8)  *277.2 [167.2]* | 160.6 (159.8)  *111.4 [60-214.3]* | t_474_ = -14.97, p < .001 |
| Weekday |  | 164.3 (161.7)  *120 [60-240]* |  |
| Weekend |  | 217.8 (200.2)  *180 [75-300]* |  |
| Entertainment | 20.4 (44.8)  *2 [0.02-18.86]* | 106.5 (129.4)  *68.57 [15-150]* | t_494_=14.86, *p* < .001 |
| Games | 30 (42.6)  *15.51 [1.86-42.37]* | 60.2 (125.6)  *10.71 [0-68.57]* | t_494_=5.32, *p* < .001 |
| Social | 132.1 (124.5)  *103.04 [27.23-204.12]* | 163.2 (226.1)  *94.29 (30-205.71)* | t_494_=3.58, *p* < .001 |

Notes: Data here are restricted to Android users only. Participants were queried separately on single- and multiplayer gaming on their phones, their streaming of videos and shows, text messaging, video chatting, and social media use. In order to better match overarching summary categories as created in Table 1, participants queried responses were collapsed as necessary (single and multiplayer into “Games”; texting, video chatting, and social media into “Social”; streaming matched “Entertainment”).

**Figure S1. Histogram of Average Daily Keystrokes Across All Participants**


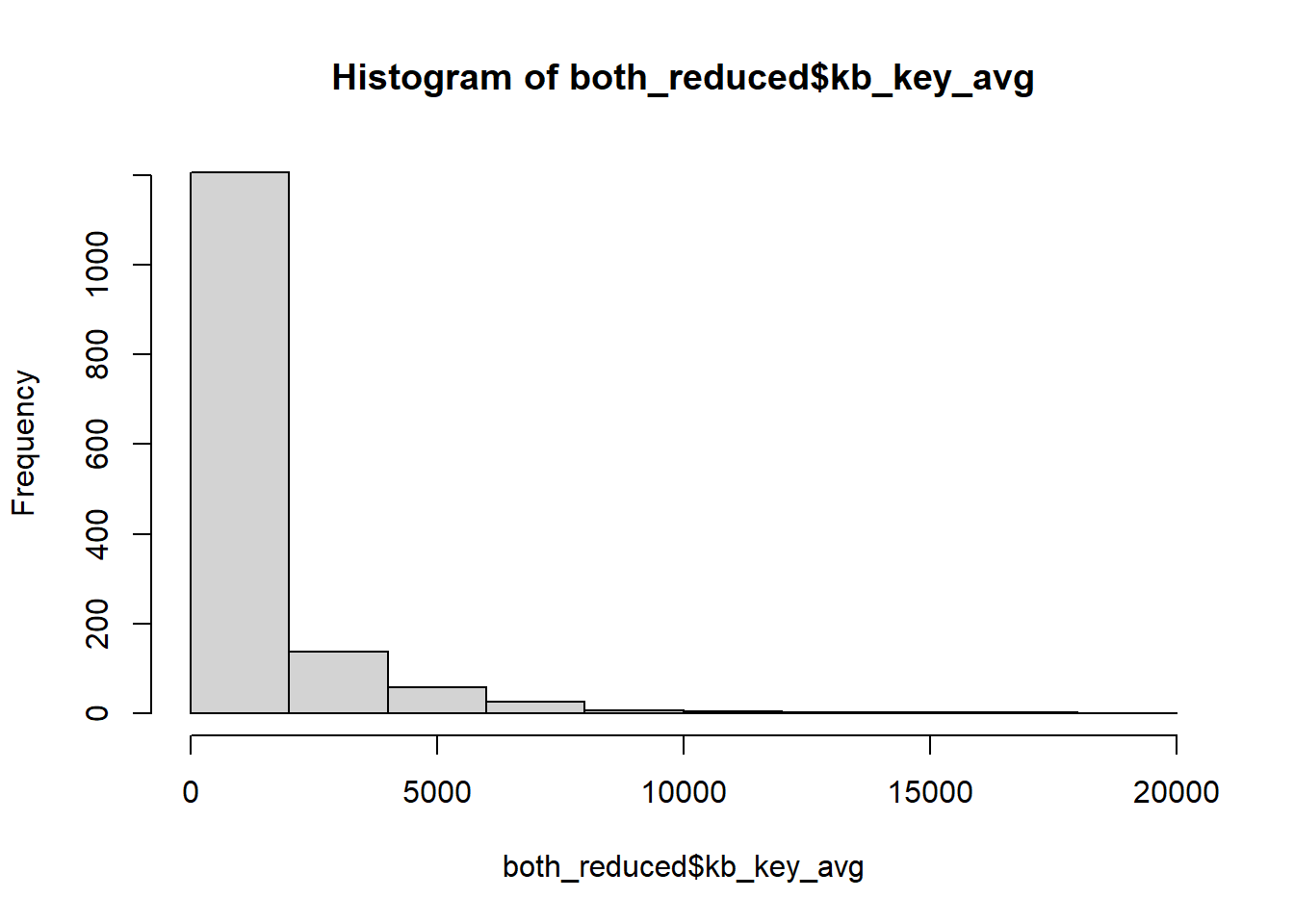


The x-axis represents the number of average daily keystrokes, and the y-axis represents prevalence (frequency) of that rate of use. Displayed data is from both iOS and Android users.

**Figure S2. Histogram of Average Daily App Use in Android Users**


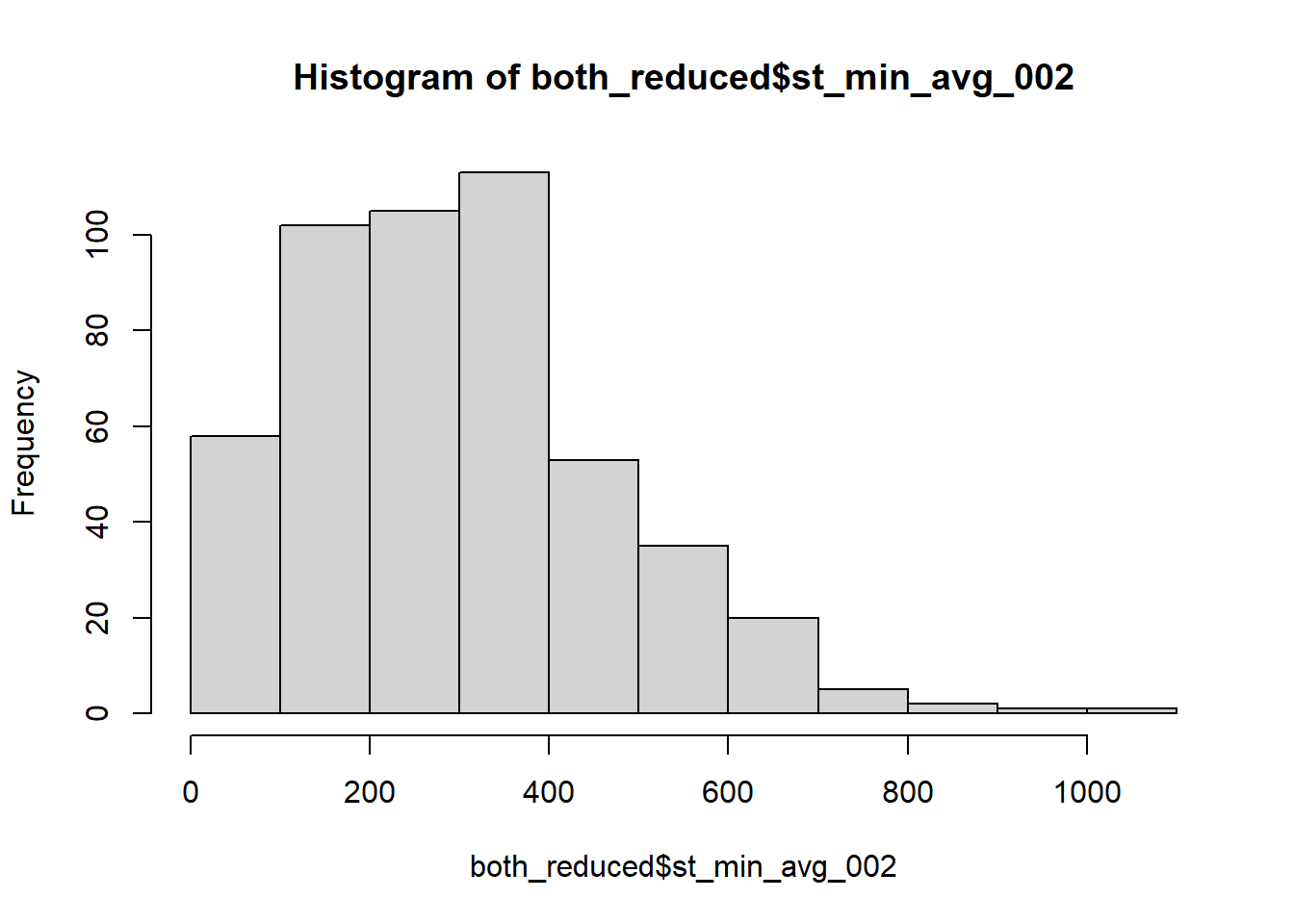


The x-axis represents the number of average daily app use (minutes), and the y-axis represents prevalence (frequency) of that rate of use. Displayed data is from Android users.

**Figure S3. Histogram of Average Self-Reported Smartphone Use (minutes) Across All Participants**


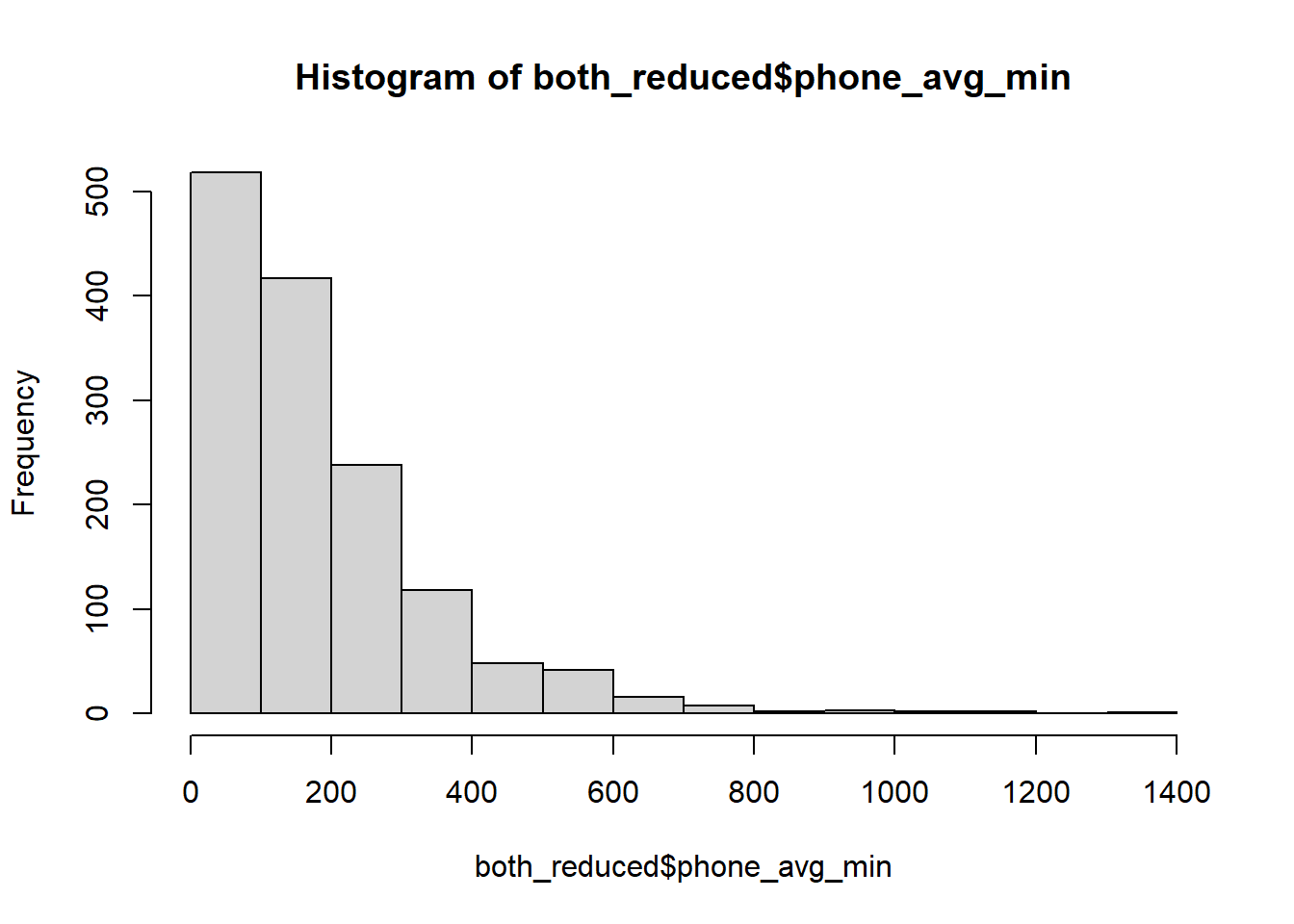


The x-axis represents the average time reported of daily smartphone use (minutes), and the y-axis represents prevalence (frequency) of that rate of use. Displayed data is from both Apple and Android users.

**Table S4. Variability and Reliability of Screen Time Measures.**

| Measure | ICC |
| --- | --- |
| Keystrokes | 0.58 (0.56,0.60) |
| Keyboard seconds | 0.55 (0.53,0.57) |
| Passively measured Smartphone Use | 0.51 (0.48,0.54) |

Notes: ICC = intraclass correlation coefficient (two-way single random raters)

**Table S5. Correlations between Self-Report and ABCD-EARS measures within Android Users**

| **Category** | **Average Daily Keystrokes: Average Daily App Use** | **Average Daily Keystrokes: Self-Reported Smartphone Use** | **Average Daily App Use: Self-Reported Smartphone Use** |
| --- | --- | --- | --- |
| Books | 0.25*** |  |  |
| Business | 0.34*** |  |  |
| Tools/Utilities | 0.12** |  |  |
| Education | 0.74*** |  |  |
| Entertainment | 0.22*** | 0.04 | 0.17*** |
| Finance | 0.27*** |  |  |
| Food | 0.52*** |  |  |
| Games | 0.51*** | 0.04 | 0.12* |
| Art | 0.42*** |  |  |
| Health | 0.23*** |  |  |
| Lifestyle | 0.33*** |  |  |
| Music | 0.23*** |  |  |
| Maps | 0.25*** |  |  |
| News | 0.20*** |  |  |
| Medical | 0.07 |  |  |
| Photography | 0.31*** |  |  |
| Productivity | 0.13** |  |  |
| Shopping | 0.80*** |  |  |
| Social | 0.55*** | 0.25*** | 0.48*** |
| Sports | 0.70*** |  |  |
| Travel | 0.15*** |  |  |
| Weather | 0.10* |  |  |
| Total Use | 0.33*** | 0.21*** | 0.35*** |

Correlations between alternative measures of smartphone use by category in Android users. Keystrokes = average daily keystrokes, Self-Reported Smartphone Use = self-reported daily average minutes of smartphone use, Average Daily App Use = average minutes of passively measured smartphone application use.

* = *p <* .05; ** = *p <* .01; *** = *p <* .001

**Table S6. Correlations between Self-Report and ABCD-EARS measures within Apple Users**

| **Category** | **Average Daily Keystrokes: Self-Reported Smartphone Use** |
| --- | --- |
| Entertainment | 0.10** |
| Games | 0.08* |
| Social | 0.02 |
| Total Use: | 0.13** |

Correlations between alternative measures of smartphone use by category in iOS users. Keystrokes = average daily keystrokes, Self-Reported Smartphone Use = self-reported daily average minutes of smartphone use. Average Daily App Use was unavailable for iOS users due to restrictions on data collection by third party applications.

* = *p <* .05; ** = *p <* .01; *** = *p <* .001

**Figure S4. Bland-Altman plot of the relationship between Average Daily App Usage and Self-Reported Smartphone Use.**


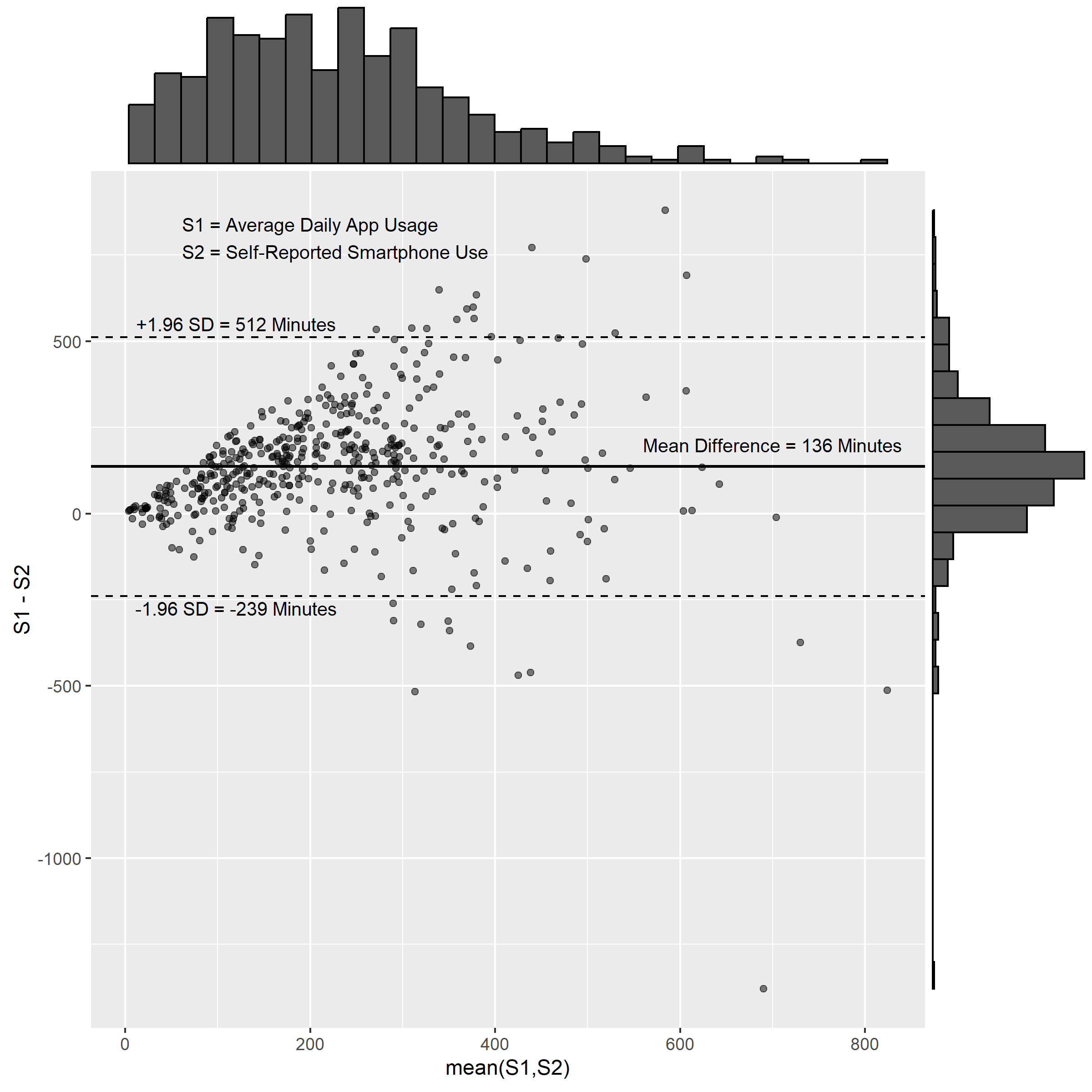


Bland-Altman plot displaying the mean of Average Daily App Usage and Self-Reported Smartphone Use on the X-axis and their difference on the Y axis. Marginal histograms represent the number of observations at each value of mean(S1,S2) and S1 – S2.

**Table S7. Average App Use by Sex**

| **Category** | **Females**  **Mean (SD)** | **Males**  **Mean (SD)** | **p-value** |
| --- | --- | --- | --- |
| Social | 02:39:40 (02:17:02) | 01:51:38 (01:50:18) | <.001 |
| Photography | 00:56:36 (01:14:18) | 01:15:13 (01:29:53) | .02 |
| Games | 00:28:28 (00:48:25) | 00:31:12 (00:37:57) | .48 |
| Entertainment | 00:25:53 (00:53:53) | 00:16:25 (00:36:33) | .02 |
| Tools/Utilities | 00:12:22 (00:15:44) | 00:13:43 (00:36:48) | .05 |
| Books | 00:11:42 (00:47:07) | 00:00:46 (00:04:53) | <.001 |
| Music | 00:07:24 (00:21:05) | 00:05:20 (00:30:26) | .40 |
| Business | 00:00:45 (00:03:22) | 00:04:53 (00:03:35) | .86 |
| Productivity | 00:03:48 (00:26:25) | 00:00:37 (00:02:00) | .04 |
| Lifestyle | 00:03:17 (00:11:05) | 00:00:19 (00:02:03) | <.001 |
| Shopping | 00:01:28 (00:03:36) | 00:00:51 (00:02:30) | .02 |
| Sports | 00:00:01 (00:00:06) | 00:00:45 (00:04:43) | .03 |
| Art | 00:00:50 (00:03:06) | 00:00:05 (00:00:34) | <.001 |
| Medical | 00:00:23 (00:00:30) | 00:00:29 (00:01:13) | .23 |
| Travel | 00:00:27 (00:02:15) | 00:00:26 (00:02:51) | .99 |
| Food | 00:00:21 (00:02:06) | 00:00:10 (00:00:44) | .18 |
| News | 00:00:03 (00:00:26) | 00:00:12 (00:01:23) | .15 |
| Weather | 00:00:02 (00:00:07) | 00:00:04 (00:00:31) | .36 |
| Maps | 00:00:01 (00:00:02) | 00:00:03 (00:00:30) | .17 |
| Total App Use | 05:25:06 (02:56:06) | 04:29:54 (02:47:05) |  |
| **Category** | **Female, mean (SD)** | **Male, mean (SD)** |  |
| Unique Apps Used per Day | 2.95 (1.63) | 2.82 (1.39) |  |
| Days with at least one recorded App Use | 20.23 (4.36) | 19.85 (5.17) |  |

Average time per day spent using each application category recorded via the ABCD-EARS app usage data in male and female Android users. Times are in hour, minute, second format.

**Table S8. Average Daily Keystrokes by App Category and Operating System**

| **Category** | **Android Users, Mean (SD)** | | **iOS Users, Mean (SD)** |
| --- | --- | --- | --- |
| Social | | 1062.20 (1976.47) | 519.66 (995.08) |
| Photography | | 22.86 (53.30) | 387.87 (976.75) |
| Tools/Utilities | | 32.17 (43.48) | 54.78 (73.98) |
| Games | | 38.88 (363.37) | 14.23 (71.83) |
| Entertainment | | 7.29 (23.09) | 38.62 (111.23) |
| Productivity | | 15.25 (109.01) | 28.09 (127.43) |
| Music | | 8.05 (78.68) | 6.15 (17.65) |
| News | | 0.02 (0.23) | 5.83 (70.85) |
| Lifestyle | | 5.63 (46.16) | 2.29 (14.15) |
| Books | | 2.99 (32.54) | 4.92 (78.02) |
| Shopping | | 4.37 (13.46) | 3.77 (24.34) |
| Business | | 1.36 (9.60) | 0.57 (5.83) |
| Travel | | 0.70 (4.54) | 0.09 (1.02) |
| Maps | | 0.003 (0.08) | 0.56 (2.29) |
| Food | | 0.49 (2.33) | 0.21 (1.39) |
| Sports | | 0.27 (2.34) | 0.28 (3.09) |
| Art | | 0.16 (1.75) | 0.11 (2.24) |
| Medical | | 0.03 (0.39) | 0.13 (3.87) |
| Weather | | 0.01 (0.12) | 0.08 (0.50) |
| **Category** | | **Android Users, Mean (SD)** | **iOS Users, Mean (SD)** |
| Unique Apps Used per Day | | 1.28 (0.93) | 1.52 (1.24) |
| Days with at least one recorded Keyboard Use | | 17.42 (6.98) | 10.43 (7.2) |

Average keystrokes per day spent using each application category recorded via the ABCD-EARS keyboard data in Apple iOS and Android users.

**Figure S5.** **Average daily app use for gender identity: nonbinary (another gender; pink), girl (blue), boy (green) for Android-using participants.**

**
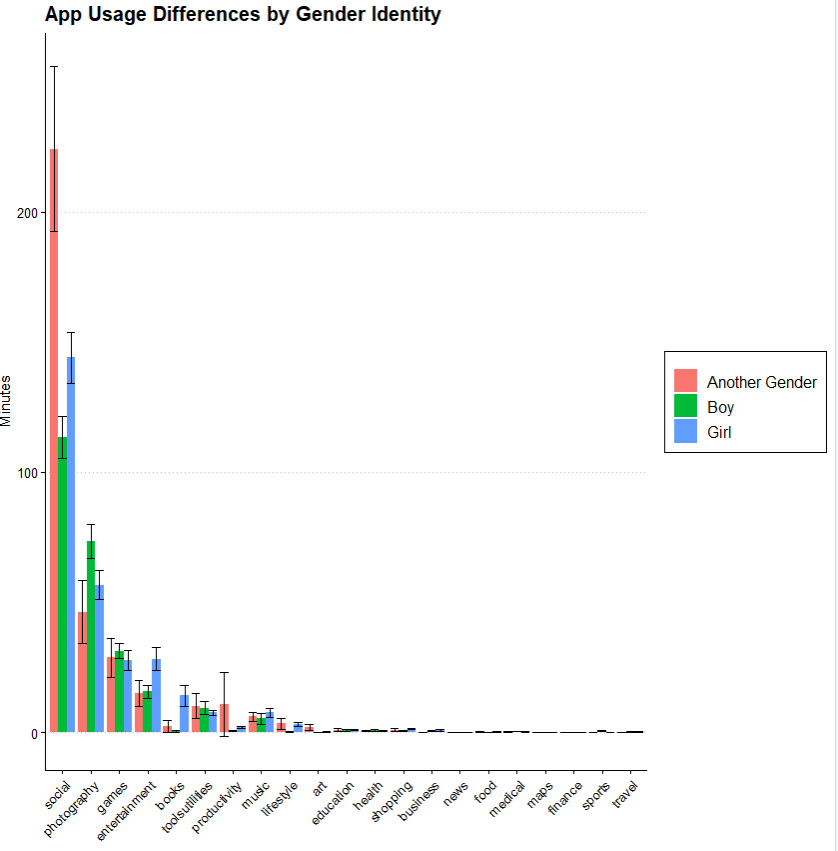
**
